# The selection of a surgical strategy for the treatment of adult degenerative scoliosis with "pear-shaped" decompression under open spinal endoscopy

**DOI:** 10.1038/s41598-024-67003-y

**Published:** 2024-07-11

**Authors:** Hongyan Wang, Xin Liu, Yuefei Li, Jiabin Ren, Zhaozhong Sun, Ning Sun, Rui Li

**Affiliations:** 1 Pain Treatment Department, BinZhou Medical University Hospital, Add: No. 522, Third Huanghe Road, BinCheng District, BinZhou City, 256603 ShanDong Province China; 2 Spinal Surgery Department, BinZhou Medical University Hospital, Add: No.522, Third Huanghe Road, BinCheng District, BinZhou City, 256603 ShanDong Province China

**Keywords:** OSE, Spinal deformity, Minimally invasive, Decompression, Surgical strategy, Outcomes research, Chronic pain, Neurosurgery

## Abstract

The prognoses of patients who undergo open spinal endoscopy (OSE) decompression significantly differ by scoliosis type and symptom despite the use of uniform standards and procedures for the decompression surgery. These differences may be directly related to the selection and formulation of surgical strategies but their cause remains unclear. The aim of this study was to verify and evaluate the efficacy of the "Symptom, Stenosis and Segment classification (SSS classification)" in determining an appropriate surgical strategy and to analyze the differences in the outcomes of different patients after receiving the selected surgical strategy. The results of this study ultimately provide a theoretical basis for the specific optimization of surgical strategies guided by the "SSS classification". This work was a retrospective study. We reviewed 55 patients with scoliosis and spinal stenosis who underwent "pear-shaped" decompression under OSE from May 2021 to June 2023 treated by our surgical team. To classify different types of patients, we defined the "SSS classification" system. The permutation and combination of subtypes in Symptom (including three subtypes: Convex = v, Concave = c and Bilateral = b), Stenosis (including three subtypes: Convex = v, Concave = c and Bilateral = b), and Segment (including two subtypes: Edge = e and Inside = i) yields 18 possible types (details in Table 1) in this classification system. To classify different types of surgeries, we also defined the operation system. The VAS Back and VAS Leg scores after surgical treatment were significantly lower in all patients 3 months after surgery than before surgery. (**P < 0.05). The Svve type accounted for the greatest proportion of patients (62.50%) in the VAS back remission group, and the Scce type accounted for the greatest proportion (57.14%) in the VAS back ineffective group. According to the VAS leg score, the percentage of patients in whom Svve was detected in the VAS leg remission group reached 60.87%, and the percentage of patients in whom Svve was detected in the VAS leg ineffective group reached 44.44%. Svve accounted for the greatest proportion of cases (61.22%) in the JOA-effective group, and Scce accounted for the greatest proportion of cases (50.00%) in the JOA-ineffective group. In the JOA-effective group, the Ovv type accounted for the greatest proportion (up to 79.59%), while in the JOA-ineffective group, Occ and Ovv accounted for 50.00% of the cases each. The proportions of Svve type were the highest in the healthy group (up to 60.00%) and the ODI-effective group (up to 50.00%). The Ovv type accounted for the greatest proportion of patients in the ODI-effective group (up to 80.00%), and the Occ type accounted for the greatest proportion of patients in the ODI-ineffective group (up to 60.00%). Most of the surgical plans formulated by the "SSS classification" method were considered appropriate, and only when the symptoms of patients were located on the concave side did the endoscopic decompression plan used in the present study have a limited ability to alleviate symptoms.

## Introduction

A large proportion of patients with lumbar spine diseases have degenerative scoliosis^1^. The clinical symptoms of most of these patients have little relation to spinal deformity and stability, and many patients experience lower limb pain and nerve dysfunction caused by spinal stenosis. For patients with mild scoliosis, especially elderly patients, the literature has confirmed that complete decompression alone can achieve a therapeutic effect^2,3^. Notably, the development of endoscopic technology has made minimally invasive interlaminar decompression more efficient and safer^4–6^. Currently, minimally invasive interlaminar decompression under endoscopy is an effective and reliable technique for the treatment of degenerative scoliosis and has been well developed. With the development and diversification of technologies, the available reliable treatment methods include traditional 10-mm endoscopy^7,8^, open spinal endoscopy (OSE) and unilateral biportal endoscopy (UBE). Among these methods, open spinal endoscopy (OSE) has been developed and promoted in recent years. Given the advantages of this endoscopic technology, tissues can be viewed through a high-definition endoscope in water, and the use of separate surgical instruments enables OSE to have a wider scope and be more efficient. With appropriate training, we can use OSE technology to perform "pear-shaped" decompression, thus allowing minimally invasive decompression to be performed in more complex situations in patients with lateral curvature and preserving a greater degree of spinal stability. As this technology continues to develop, we found that the prognoses of patients undergoing OSE decompression significantly differ by scoliosis type and symptom despite the use of uniform standards and procedures for the decompression surgery. We believe that these differences may be directly related to the selection and formulation of surgical strategies, which are generally affected by multiple complex factors. Notably, differences may be closely related to patients’ lateral curvature and clinical symptoms. We reviewed 55 cases of patients with degenerative scoliosis treated by "pear-shaped" decompression under OSE completed by our team from May 2022 to June 2023. The patients were classified according to their scoliosis status, clinical symptoms, stenosis side, and stenosis segment, i.e., the proposed "SSS classification". In addition to undergoing surgery, the patients were followed for 3 months after surgery. The aim of this study was to verify and evaluate the efficacy of the "SSS classification" in determining an appropriate surgical strategy and to analyze the differences in the outcomes of different patients after receiving the selected surgical strategy to ultimately provide a theoretical basis for the specific optimization of surgical strategies guided by the "SSS classification".

## Materials and methods

### Participant

We reviewed 55 patients with scoliosis with spinal stenosis who underwent "pear-shaped" decompression under OSE from May 2021 to June 2023 treated by our surgical team.

This study was approved by the Research Ethics Committee of BinZhou Medical University Hospital in BinCheng, BinZhou, PR China.

All operations were performed by the same surgeon. The reference inclusion criteria were as follows: (1) a coronal Cobb angle greater than 0° and less than 30° in patients with degenerative scoliosis; (2) unilateral extremity radicular pain with or without intermittent claudication; (3) single-level lumbar spinal stenosis, with imaging findings consistent with the symptoms; (4) ineffective conservative treatment for more than 3 months; and (5) decompression of the spinal canal using OSE. The exclusion criteria were as follows: (1) severe low back pain; (2) a coronal Cobb angle greater than 30°; (3) anterior lumbar spondylolisthesis present in the sagittal plane; (4) global lumbar spine instability; and (5) loss to follow-up.

To classify different types of patients, we defined the "SSS classification" system as follows.

1. Symptoms: ① Convex = v, ② Concave = c, or ③ Bilateral = b.
2. Stenosis: ① Convex = v, ② Concave = v, or ③ Bilateral = b
3. Segment: ① Edge = e or ② Inside = i

The classification of patients is determined by the combination of specific symptoms, stenosis, and segment subtypes. The permutation and combination of symptom, stenosis, and segment subtypes yields 18 possible scoliosis types in this classification system. For instance, if a patient presents with symptoms such as pain or numbness on the convex (abbreviated as ‘v’) side, while the stenosis of the spinal canal is located on the concave (abbreviated as ‘c’) side and involves the edge (abbreviated as ‘e’) of scoliosis, it should be classified as the subtype Svce.

**Figure 1:**
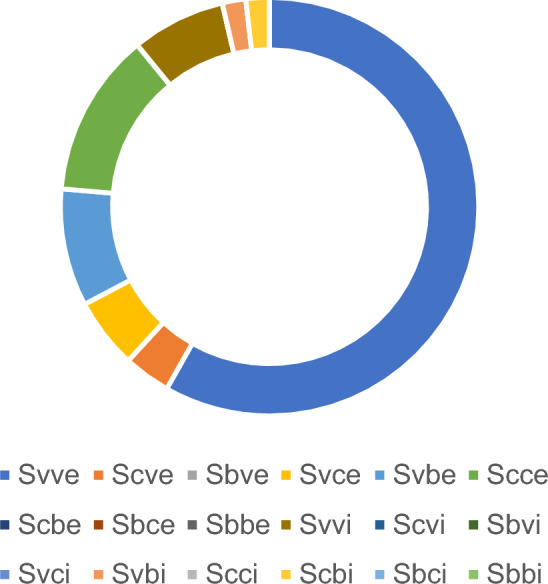
Cases in the "SSS classification" system.

Fifty-five patients were numbered 1–55. First, each patient was classified using the "SSS classification" system, labeled and categorized, as shown in Table 1 and Fig. 1.

**Table 1:** Cases in the "SSS classification" system.

Classification	Cases	Classification	Cases
Svve	32	Svvi	4
Scve	2	Scvi	0
Sbve	0	Sbvi	0
Svce	3	Svci	0
Svbe	5	Svbi	1
Scce	7	Scci	0
Scbe	0	Scbi	1
Sbce	0	Sbci	0
Sbbe	0	Sbbi	0

To classify different types of surgeries, we also defined the operation system (abbreviated as ‘O’) as follows:

1. Approach: ① Convex = v or ② Concave = c
2. Decompression: ① Convex = v, ② Concave = c, or ③ Bilateral = b.

If a patient underwent surgery via the convex side approach (abbreviated as ‘v’) and underwent bilateral decompression (abbreviated as ‘b’), the surgery was classified as the operation subtype Ovb. The case numbers of each operation type with different approach or decompression scales are shown in Table 2.

**Table 2:** Cases in the "Operation classification" system.

Classification	Cases	Classification	Cases
Ovv	42	Ocv	0
Ovb	5	Ocb	1
Ovc	0	Occ	7

In addition to considering the difficulty coefficient of various surgical strategies, we referred to the following principles to guide the choice of surgical strategy: ① The symptomatic side should be considered first in determining the surgical approach side; ② the narrow side, as shown by imaging, plays a secondary role in determining the side of the surgical approach; ③ in cases of conflict, the surgical approach with the lowest difficulty coefficient and the highest safety should be selected.

Here, basic strategies for the treatment of simple endoscopic decompression were developed for each patient: ① For patients with convex symptoms, regardless of the concavity of stenosis on imaging, decompression was performed through a convex-side interlaminar approach. ② Only when the patient had symptoms on the concave side and imaging indicates stenosis on the concave side was decompression performed with an interlaminar approach on the concave side. ③ For patients with concave-side symptoms and imaging evidence of convex or bilateral spinal canal stenosis, bilateral decompression through a convex-side interlaminar surgical approach was generally selected; however, a small number of patients chose to undergo surgery with a convex-side interlaminar approach for simple concave-side decompression.

### Surgical technique

**Figure 2:**
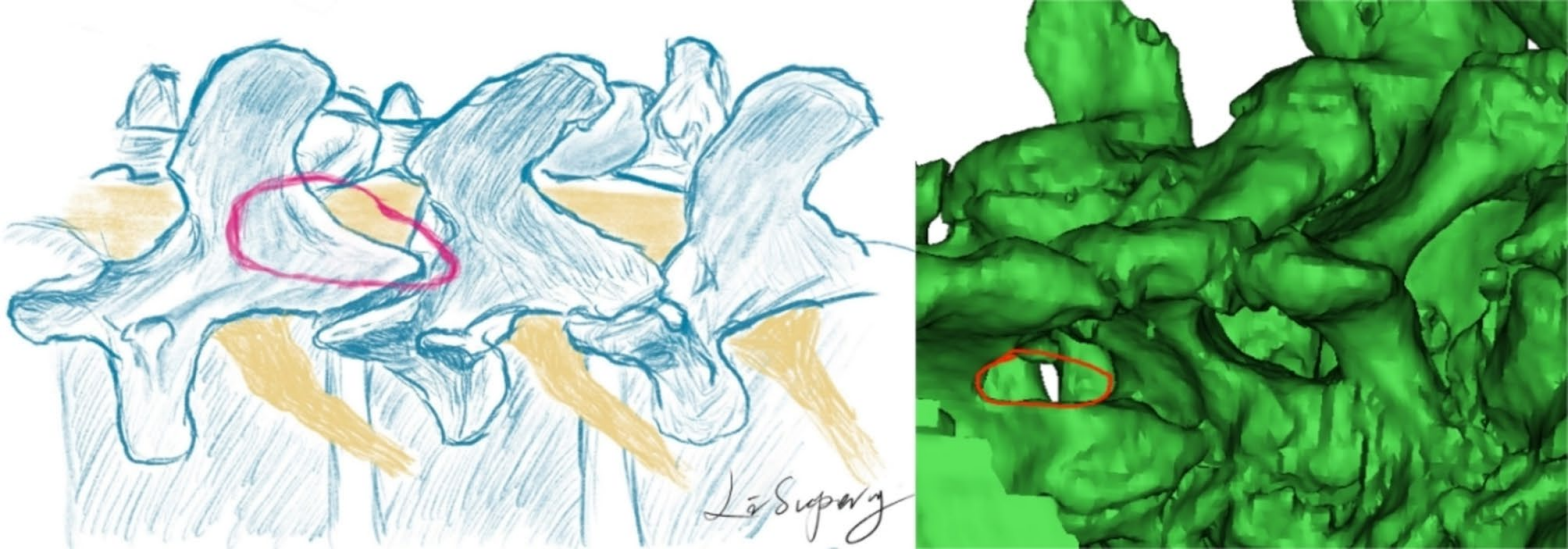
Scope of "pear-shaped" decompression.

Lumbar decompression under OSE is a standardized surgical technique. Although the condition of patients differed, the surgical procedure was the same. After receiving general anesthesia, the patient was placed in a prone position with lumbar spine flexion and abdominal suspension. The location of the spinous process was recorded on the body surface, and the spatial morphology of the spinous process was initially marked on the body surface. Lateral fluoroscopy was used to determine the vertebral space initially, and a 1.5-cm longitudinal incision was made at the lateral spinous process at the location of the intervertebral surface. The surgeon aimed to make the incision completely longitudinal through the deep fascia and to position the dilating cannula at the lower margin of the upper lamina in the laminar space and gradually expand it. During the dilation process, the cannula edge could be used to clear the muscle attachment point. After entering the lens, the laminar space was cleared, and the lower margin of the upper lamina and the junction of the spinous root were ground by a ball grinding drill. First, from the interlaminar space to the head end to the endpoint of the ligamentum flavum and then from the tail end to the deep inferior laminae, the ligamentum flavum was completely detached; then, after removal of the collateral ligamentum flavum, the dural membrane could be clearly exposed, and the 30° endoscopic angle could be adjusted to perform "pear-shaped" potential decompression (Fig. 2). When contralateral decompression was needed, the surgeon aimed to temporarily retain the ligamentum flavum on the approach side and ground and drilled through the root of the spinal process across the centerline to the contralateral side, grinding to the medial edge of the supreme articular process until the contralateral dural membrane and nerve root could be exposed after the contralateral ligamentum flavum was removed. Finally, the ligamentum flavum on the approach side could be resected. The criteria for concluding the decompression surgery were as follows: ① The range of dorsal decompression was from the lateral margin of the dura to the shoulder of the nerve root to the internal opening of the foramina. ② Obvious compression of the ventral nerve root was not evident. (3) When the nerve root was removed, it was relaxed, and no obvious tension was observed at the distal end. After the water pressure decreased, the nerve root and dural membrane pulsated. ④ The nerve root probe could successfully enter the internal opening of the foramen and explore outwardly. Generally, no indentured drainage tube was placed in the incision, and sterile excipients were used to cover the incision after a single needle suture. Patients were allowed to attempt ambulation with a brace 6–7 days after surgery.

### Patient follow-up

The VAS score, JOA score and ODI were measured and recorded for each patient before surgery, 1 day after surgery, 1 month after surgery and 3 months after surgery. The Cobb angle, pelvic incidence angle, PI, lumbar lordosis angle, LL and PI–LL were measured before and 3 months after surgery.

Patients were grouped according to the following indicators: (1) Patients were divided according to their VAS score into a VAS remission group (score = 0–3) and a VAS ineffective group (score = 4–10). ② According to the JOA score, the patients were divided into a JOA-effective group (improvement rate ≥ 25%) and a JOA-ineffective group (improvement rate < 25%). ③ According to the ODI index, the patients were divided into an ODI-effective group at the 3-month postoperative time point (ODI ≤ 20%) and an ODI-ineffective group at the 3-month postoperative time point (ODI > 20%). First, the specific SSS types and surgical strategies used in the different groups were analyzed, and differences in the Cobb angle, PI angle, LL angle and PI-LL angle among the three groups at different time points before and after surgery were compared.

### Data analysis

The continuous variables studied are expressed as the mean ± standard deviation. A t test was used for comparisons between independent and continuous variables, and a chi-square test was used for comparisons between categorical variables. Values of P < 0.05 were considered to indicate significant differences. Statistical calculations were performed using IBM SPSS (version 23.0, IBM Corp.).

### Informed consent statement

All patients or their legal guardians provided informed consent for this study. All methods were carried out in accordance with the Declaration of Helsinki.

## Results

### Patient preoperative clinical data

**Figure 3:**
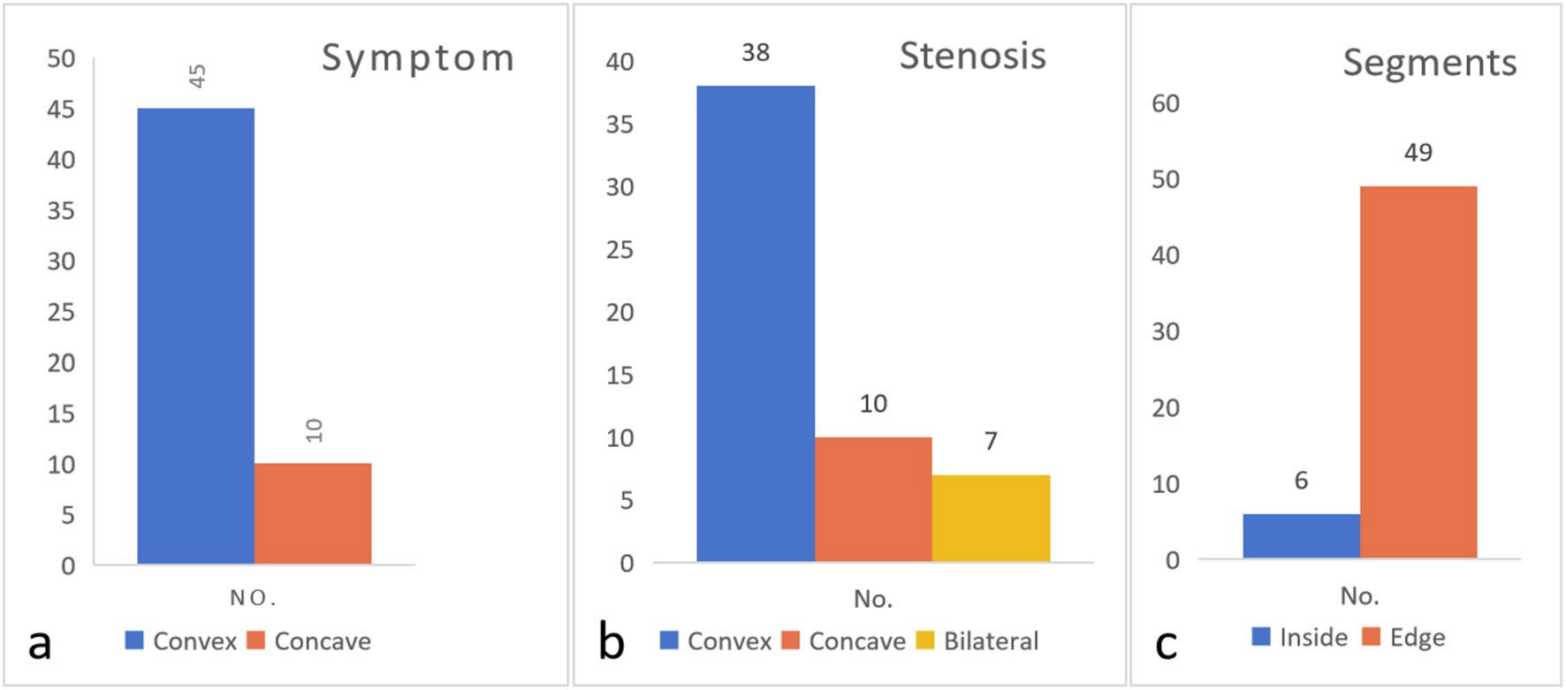
Cases in each group according to the "SSS classification" system. (**a**) Forty-five patients had symptoms on the convex side, and 10 patients had symptoms on the concave side. (**b**) Thirty-eight patients had convex stenosis, 10 patients had concave stenosis, and 7 patients had bilateral stenosis. (**c**) Six patients had stenotic segments located inside the curve, and 49 patients had stenotic segments located at the curve edge.

We reviewed 55 patients with scoliosis and spinal stenosis who underwent "pear-shaped" decompression under OSE from May 2021 to June 2023 treated by our team. The patients included 15 males and 40 females, with an average age of 51.35 ± 5.18 years. We defined the SSS method and categorized all patients by the relationships between their symptom side, stenosis side, stenosis segment location, and lateral curvature. In total, 45 patients had symptoms on the convex side, and 10 patients had symptoms on the concave side. In total, 38 patients had convex stenosis, 10 patients had concave stenosis, and 7 patients had bilateral stenosis. Six patients had stenotic segments located inside the curve, while 49 patients had stenotic segments located at the curve edge (Fig. 3). We performed surgical treatment on each patient according to the specific chosen strategies. The scope of the surgical approach and decompression is shown in the table.

### Complications

We have completed many surgical procedures for spinal canal decompression under OSE, and the surgical safety of the procedure has been confirmed. Among the 55 patients included in this study, 2 (3.64%) had asymptomatic microscopic dural tears, and 1 patient had a fracture of the lower articular process due to excessive grinding during decompression. The patient experienced unbearable lower back pain when standing up and moving after surgery and is still receiving further conservative treatment. Other complications, including anesthesia-related complications and nerve damage, were not observed.

### Evaluation of therapeutic effect

**Figure 4:**
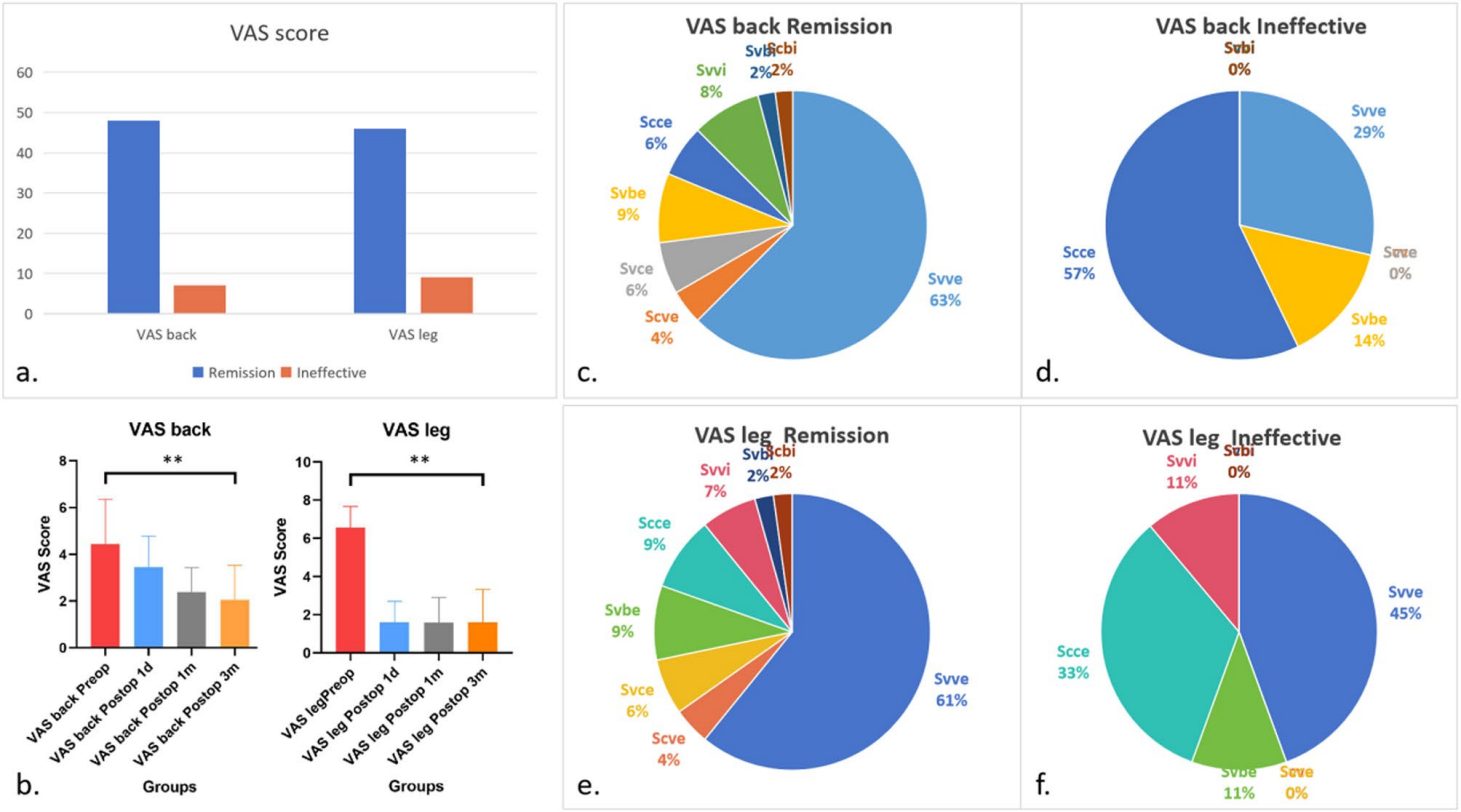
Analysis of the VAS score for the back and leg. (**a**) The VAS back score remission group included 48 patients, whereas the VAS back score ineffective group included 7 patients. The VAS leg score remission group included 46 patients, whereas the VAS leg score ineffective group included 9 patients. (**b**) The VAS back and leg scores after surgical treatment of all patients were significantly lower 3 months after surgery than before surgery (** P < 0.05). (**c**, **d**) The Svve type accounted for the highest proportion of cases (62.50%) in the VAS back remission group, and the Scce type accounted for the highest proportion (57.14%) in the VAS back ineffective group. These rates significantly differed (P = 0.0303). (**e**, **f**) Svve type curves constituted 60.87% and 44.44% of cases in the VAS leg remission group and VAS leg ineffective group, respectively. These rates did not significantly differ (P = 0.5902).

The VAS score encompasses the low back pain score and lower limb pain score and can subjectively evaluate back pain and leg pain; the VAS score can also approximately reflect the patient’s own evaluation of the surgical effect. In general, the VAS Back and VAS Leg scores after surgical treatment were significantly lower for all patients 3 months after surgery than before surgery. We believe that a VAS score lower than 3 indicates mild or reduced pain, which indicates that the therapeutic goal has been achieved; if the VAS score exceeds 4, the patient still has moderate or worse pain, and the therapeutic effect is lower than expected. Different aspects of pain should be treated differently. We first divided the patients into the VAS back remission group and VAS back ineffective group according to their low back pain status and then divided them into the VAS leg remission group and VAS leg ineffective group according to their lower extremity pain status. The VAS back remission group included 48 patients, the VAS back ineffective group included 7 patients, the VAS leg remission group included 46 patients, and the VAS leg ineffective group included 9 patients. We evaluated the relationships among the different groups, lumbar spine classifications and surgery types and found that the Svve-type subgroup accounted for the greatest proportion of patients (62.50%) in the VAS back remission group, and the Scce-type subgroup accounted for the greatest proportion (57.14%) in the VAS-back ineffective group. These percentages significantly differed (P = 0.0303). In the VAS score remission group, the Ovv type accounted for the highest proportion (up to 81.25%). In the VAS back ineffective group, the Occ type accounted for the highest proportion (up to 57.14%). These rates significantly differed (P = 0.0007). According to the VAS leg score, patients with Svve type curves constituted 60.87% of the VAS leg remission group, whereas this proportion was 44.44% in the VAS leg ineffective group. However, these rates did not significantly differ (P = 0.5902). The most common surgery type in the VAS leg remission group (80.43%) and the VAS leg ineffective group (66.67%) was Ovv, but these rates did not significantly differ (P = 0.093) (Fig. 4).

**Figure 5:**
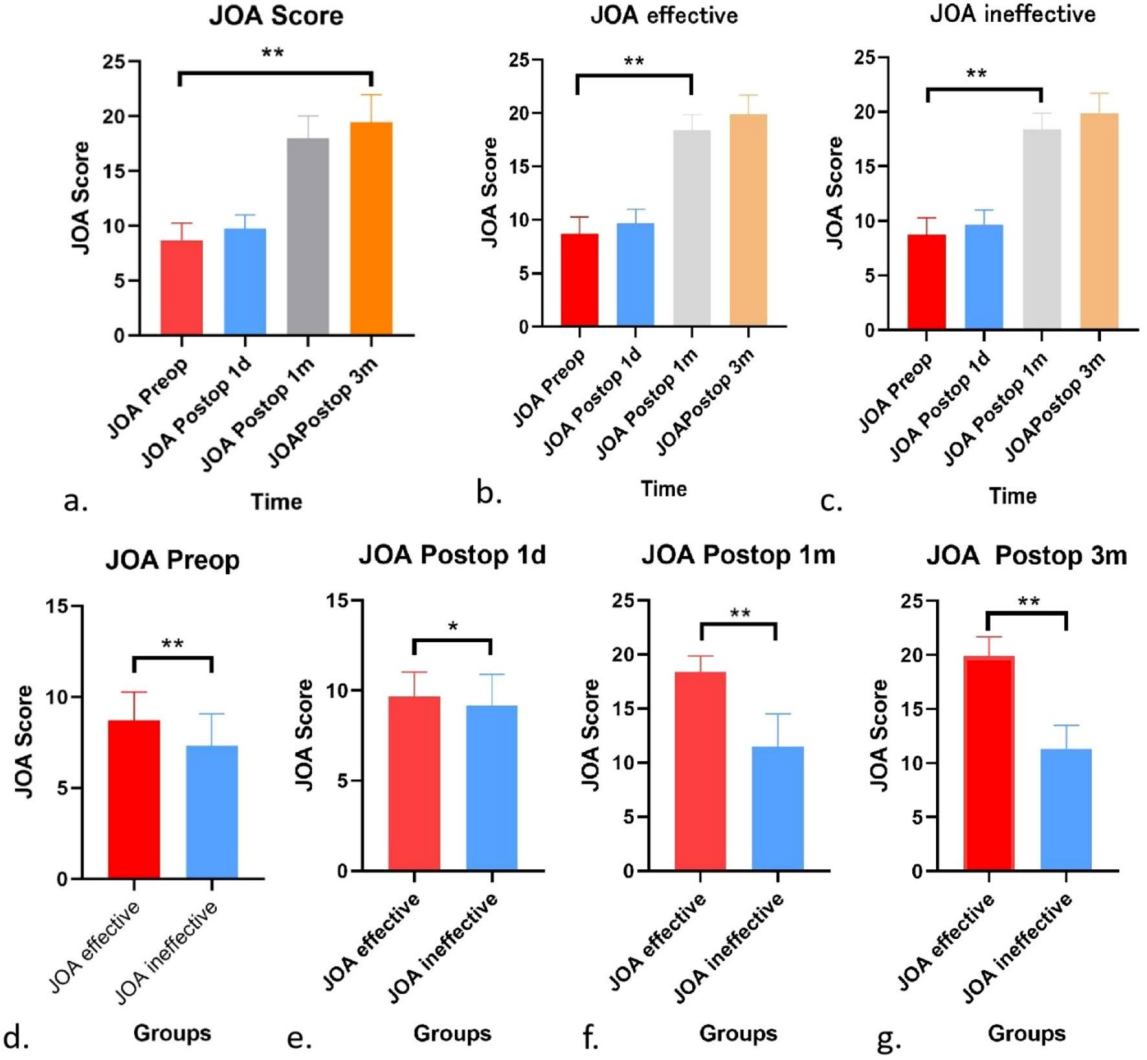
The JOA analysis in the study. (**a**–**g**) Overall, the 3-month postoperative JOA score significantly increased compared with the preoperative score (**P < 0.05). In both the effective and ineffective JOA groups, the 1-month postoperative JOA score significantly increased compared with the preoperative JOA score (**P < 0.05). A significant difference was identified between the effective and ineffective JOA groups at 1 month and 1 month after the surgery (**P < 0.05), but no significant difference was identified between these groups 1 day postsurgery (*P > 0.05).

The JOA and ODI reflect the quality of life of patients well, and the two evaluation systems have overlapping but different emphases. The JOA score can be used to evaluate the therapeutic effect of surgery based on the improvement rate. Based on the JOA analysis in the present study, most patients achieved good symptom relief (Fig. 5).

**Figure 6:**
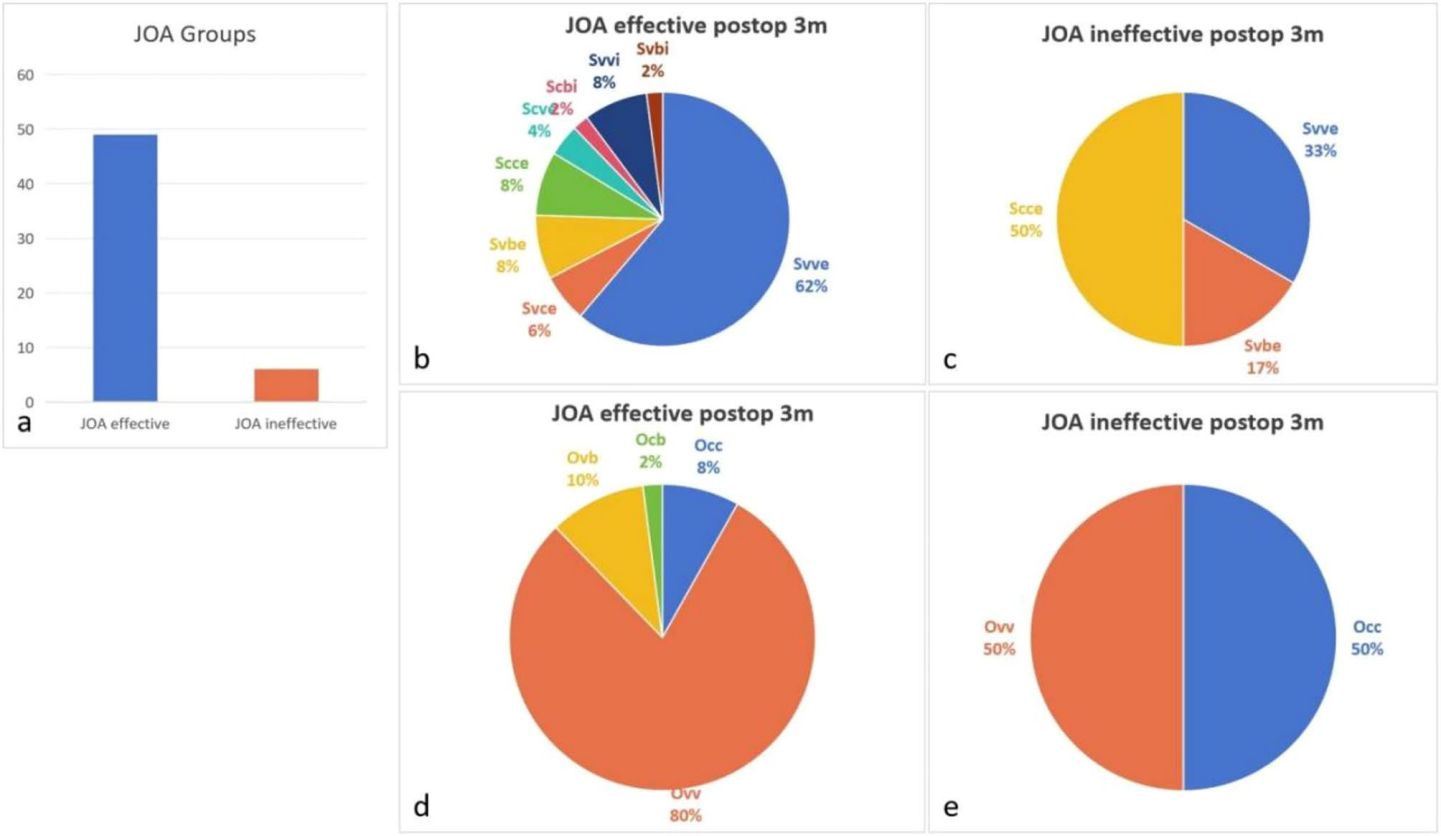
The distribution of patients in different JOA groups. (**a**) The JOA effective group included 49 patients, and the JOA ineffective group included 6 patients. (**b**, **c**) Svve accounted for the greatest proportion of cases (61.22%) in the JOA effective group, and Scce accounted for the greatest proportion of cases (50.00%) in the JOA ineffective group. These rates did not significantly differ (P = 0.1979). (**d**, **e**) Ovv surgeries were most common in the JOA effective group (up to 79.59%), whereas Occ and Ovv each accounted for 50.00% of surgeries in the JOA ineffective group. These rates significantly differed between the two groups (P = 0.0336).

We considered an improvement rate ≥ 25% an effective treatment, and an improvement rate less than 25% was considered ineffective treatment. According to the JOA improvement rate, patients were divided into a JOA effective group and JOA ineffective group; the JOA effective group included 49 patients, and the JOA ineffective group included 6 patients. The Svve type accounted for the greatest proportion of cases (61.22%) in the JOA effective group, and the Scce type accounted for the greatest proportion of cases (50.00%) in the JOA ineffective group. These rates did not significantly differ (P = 0.1979). In the JOA effective group, the most common surgery type was Ovv (up to 79.59%), whereas Occ and Ovv each constituted 50.00% of surgeries in the JOA ineffective group; these rates significantly differed between the groups (P = 0.0336) (Fig. 6).

**Figure 7:**
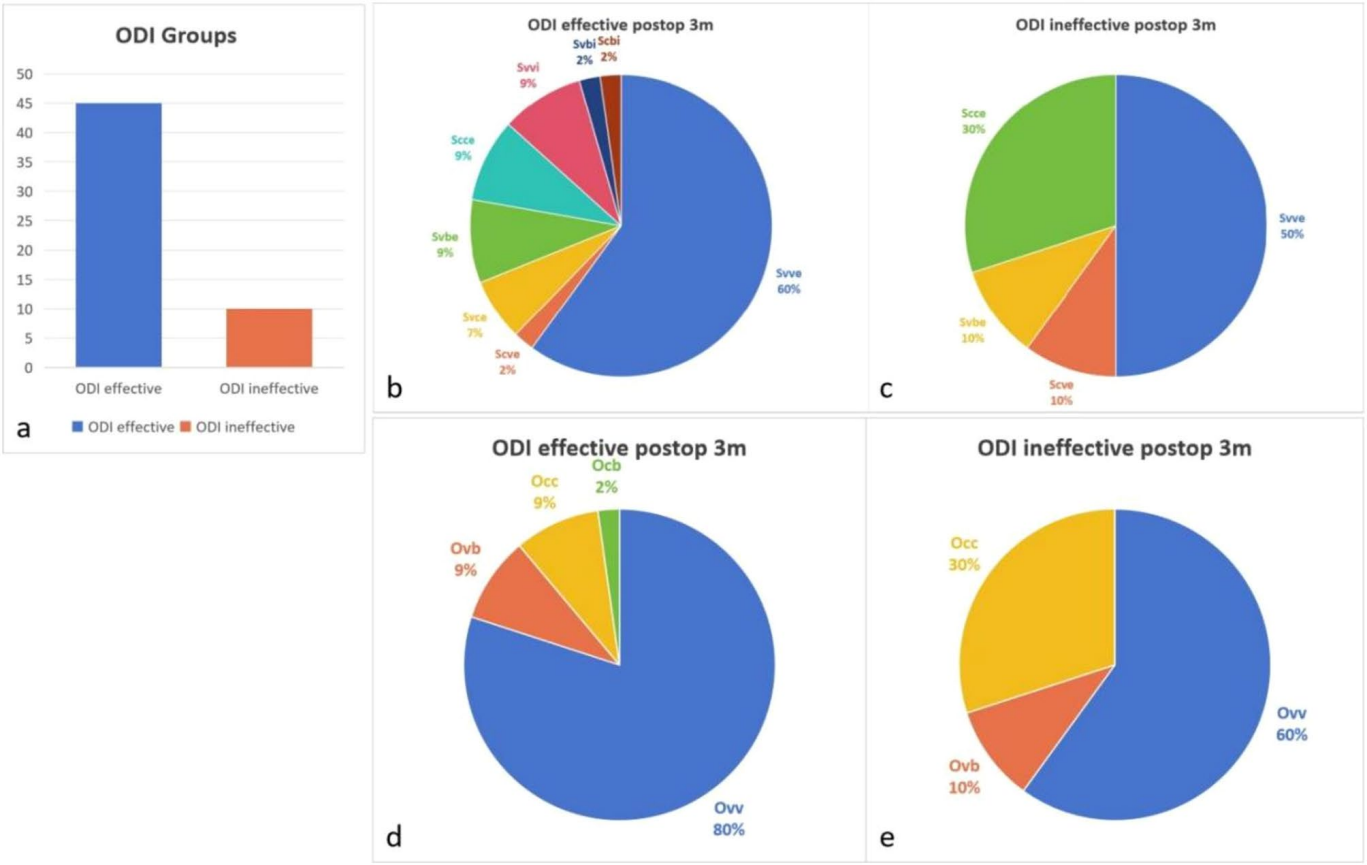
The distribution of patients in different ODI groups. (**a**) The effective ODI group included 45 patients, and the ineffective ODI group included 10 patients. (**b**, **c**) Svve type curves were most common in the healthy group (up to 60.00%) and ODI effective group (up to 50.00%). These rates did not significantly differ (P = 0.4964). (**d**, **e**) The Ovv type accounted for the greatest proportion of the ODI effective group (up to 80.00%), and the Occ type accounted for the greatest proportion of the ODI ineffective group (up to 60.00%). The proportions of surgical types in the two groups were not significantly different (P = 0.3173).

The ODI is a percentage that distinguishes the degree of dysfunction, and an ODI of at least 20% indicates the ability to maintain a high quality of life. Therefore, patients were divided into an effective ODI group (n = 45) and an ineffective ODI group (n = 10) according to whether the ODI was greater than 20%. Svve-type curves were most common in the healthy group (up to 60.00%) and the ODI effective group (up to 50.00%), and these rates did not significantly differ (P = 0.4964). Ovv surgeries were most common in the ODI effective group (up to 80.00%), whereas Occ surgeries were most common the ODI ineffective group (up to 60.00%). However, these rates did not significantly differ (P = 0.3173) (Fig. 7).

**Figure 8:**
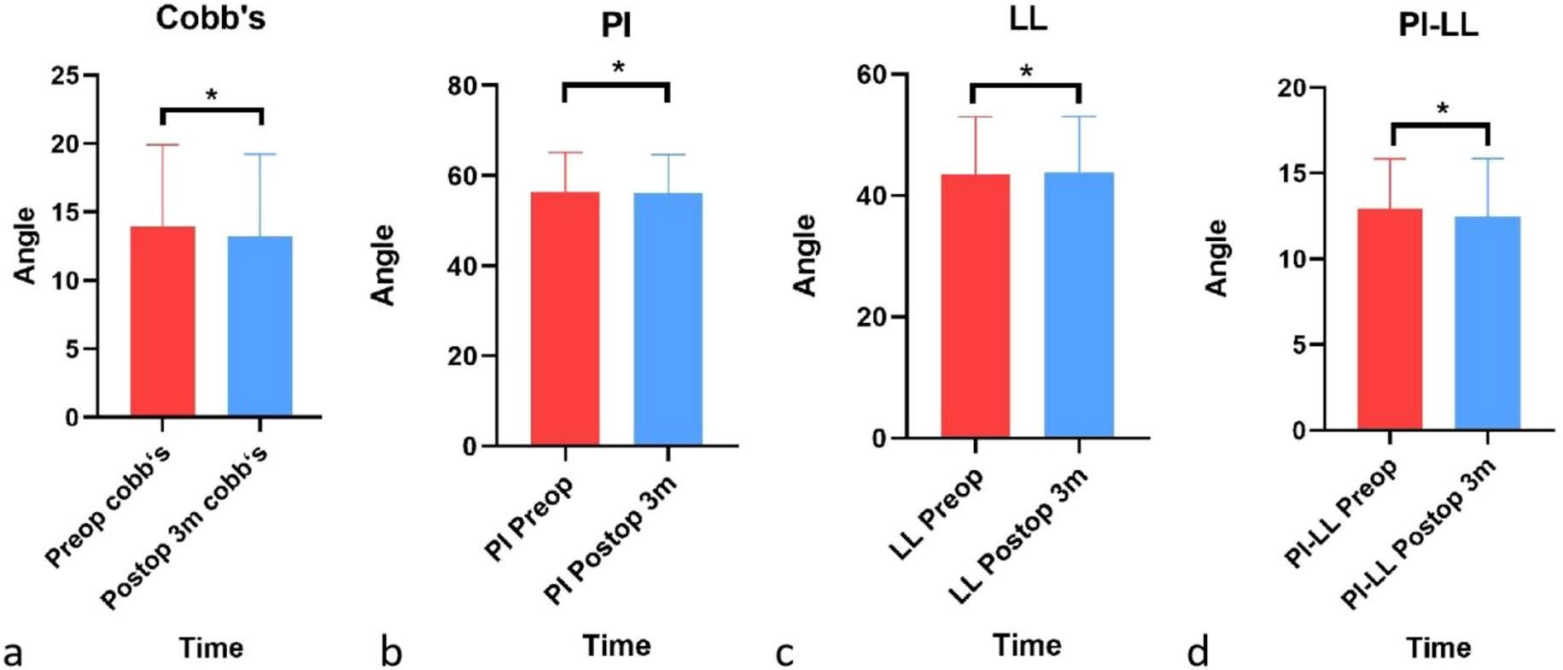
Differences in the Cobb angle, PI angle, LL angle or PI-LL angle before or 3 months after surgery. (**a**–**d**) The Cobb angle, PI angle, LL angle or PI-LL angle before and 3 months after surgery did not significantly differ (*P > 0.05).

The Cobb angle, PI angle, LL angle or PI-LL angle did not significantly differ before or 3 months after surgery (Fig. 8).

**Figure 9:**
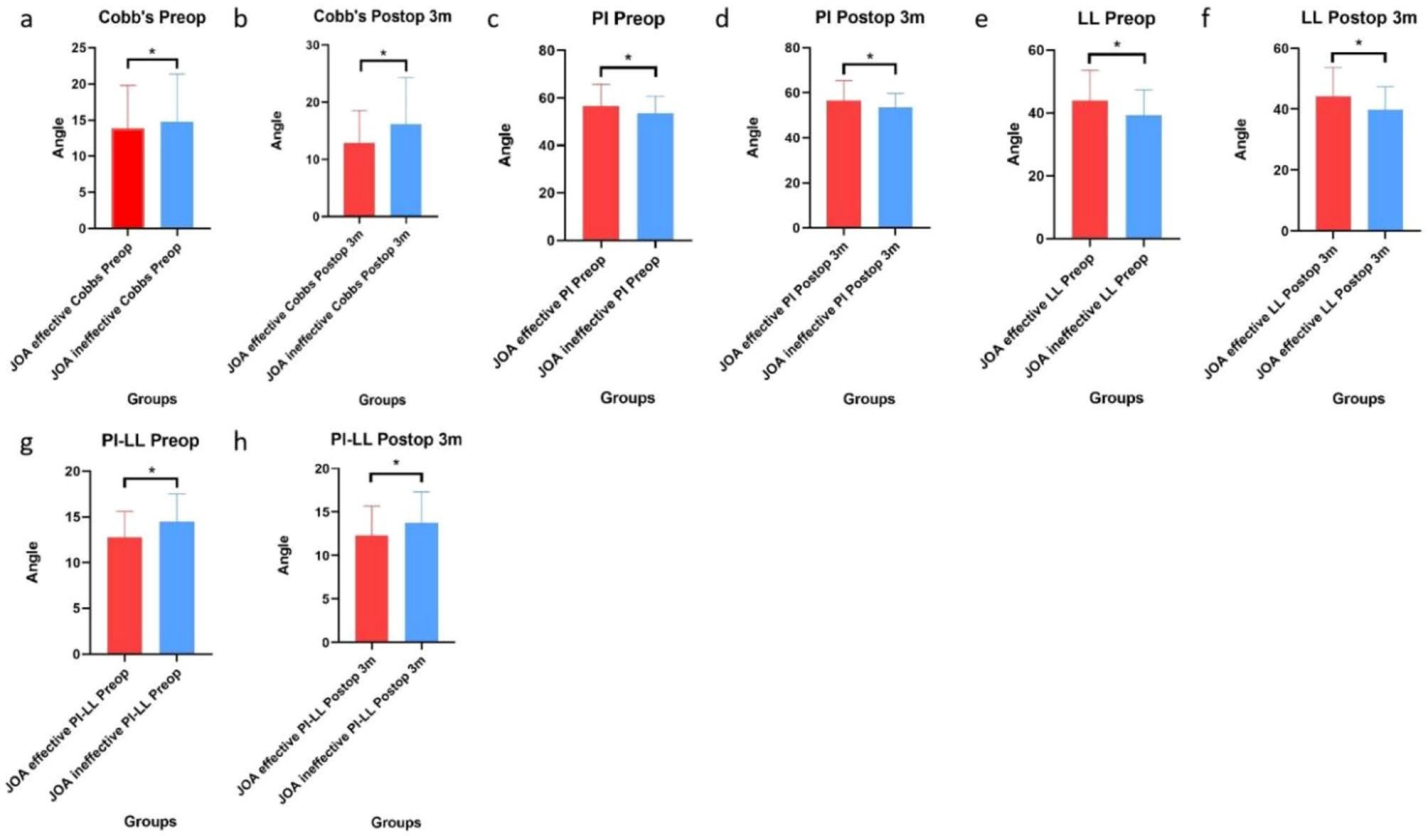
Differences in angles between the JOA effective group and the JOA ineffective group. (**a**–**h**) The Cobb angle, PI angle, LL angle or PI-LL angle before or 3 months after surgery did not significantly differ between the JOA effective group and the JOA ineffective group (*P > 0.05).

**Figure 10:**
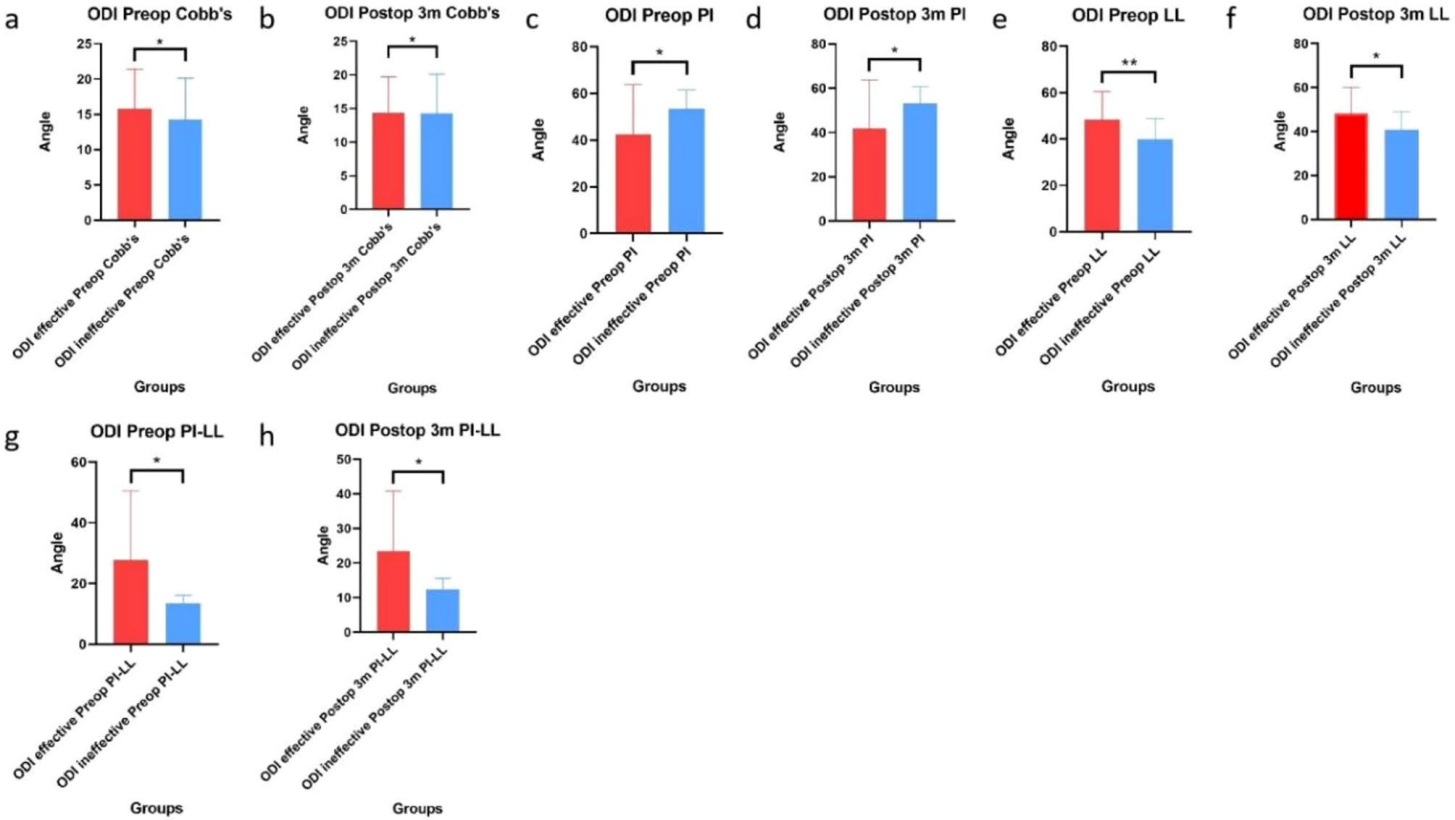
Differences in angles between the effective ODI group and the ineffective ODI group. (**a**–**h**) The Cobb angle, PI angle, LL angle or PI-LL angle before and 3 months after surgery did not significantly differ between the ODI effective group and the ODI ineffective group (*P > 0.05).

In addition, the Cobb angle, PI angle, LL angle or PI-LL angle did not significantly differ between the JOA effective group and the JOA ineffective group before or 3 months after surgery (Fig. 9). Moreover, the angles of the ODI effective group and the ODI ineffective group were similar (Fig. 10).

## Discussion

Clinically, scoliosis is common in patients with lumbar spine disorders^9^. Reduced water content and height loss of the intervertebral disc may be the first pathological changes to occur in adults with degenerative scoliosis. Alternatively, vertebral body morphological changes caused by osteoporosis may also be important pathological changes^10^. The accompanying increase in the load on the facet joints accelerates the degeneration of the spine, and the spine gradually becomes unstable and rotates, which results in scoliosis in the coronal plane^11^. When scoliosis is present, the surgical treatment plan for the patient must be carefully considered. First, the relationships between patients’ clinical symptoms and scoliosis should be fully evaluated. Moreover, the local structural changes caused by surgery should be considered when predicting whether the overall stability of the spine will improve or whether there is a greater risk of worsening the overall stress on the spine. These considerations will guide the need of treatment plans to involve scoliosis correction with internal fixation. Linke-Silva’s criteria^12^ specify intermittent clotting, less pain in the lower back, and intervertebral slippage. Patients with a 2 mm, well-balanced coronal position, Cobb angle < 30°, lack of kyphosis and/or overall imbalance may receive surgical treatment with spinal canal decompression alone. This evaluation criterion includes clinical symptoms and imaging findings of patients and is the underlying criterion for establishing the SSS classification system.

We believe that the clinical symptoms of patients must be related to scoliosis, and the side of scoliosis is also related to the side on which patients have symptoms. Current studies have also described neurogenic pain caused mainly by compression of nerve roots or dorsal root ganglia in the foraminal area on the concave side of scoliosis or neurogenic claudication caused by central spinal canal stenosis ^13^.

Based on previous studies and theoretical speculation, we initially hypothesized that the symptoms caused by scoliosis mainly occurred on the concave side of scoliosis because the foraminal area on the concave side of scoliosis compresses the nerve roots, explaining the symptoms. Surprisingly, approximately 81.82% of the patients included in this study had symptoms on the convex side of scoliosis, and only 18.18% had symptoms on the concave side of scoliosis. To avoid large errors caused by sample limitations, we also conducted expanded statistical analyses of similar patients undergoing surgery, and the results showed that this proportion was consistent with the local disease spectrum distribution. Our results suggest that patients who undergo surgery generally have mild scoliosis and are satisfied with decompression surgery only; therefore, the scoliosis is not severe enough in most cases to compress the nerve root in the concave foraminal area. In addition, the patient’s spinal morphological changes and discogenic lumbar symptoms during disc degeneration are relatively serious, and some patients also experience pain at the base of the thigh due to vertebral rotation^14^.

Previous studies and follow-up results have guided the selection of orthopedic surgery or other treatments^15,16^, but detailed guidance for simple endoscopic decompression surgery is lacking. Extensive and systematic decompression and sequence reconstruction are easily achieved during orthopedic surgery, while simple decompression of the spinal canal under endoscopy requires a more subtle judgment of the location of selected segments and the decompression range. During simple endoscopic decompression, interlaminar bone destruction is necessary, and grinding the medial edge of some inferior and superior articular processes may also be necessary. Lateral bending conditions and surgical plans may vary to achieve decompression. We hypothesized that this differentiated selection may have a distinct and lasting impact on the outcome of surgery and the long-term progression of scoliosis. The main purpose of this study was to evaluate the appropriateness of our surgical protocol for patients with different types of scoliosis.

Based on the VAS, JOA, and ODI results in the present study, most patients achieved good symptom relief. However, when the symptomatic side was the concave side, the VAS score suggested that the relief of lower back pain was significantly limited in patients with different types of concave side stenosis (Scce) and those who underwent concave side approach decompression (Occ), and lower limb symptoms did not significantly improve. In addition, the results of the JOA analysis indicate that the quality of life of these patients is limited, perhaps because the bone destruction necessary during surgery may further reduce the local structural strength of the concave side of the spine in this type of patient. Therefore, they are more likely to have residual lumbar symptoms, which affect the surgical prognosis. Surgical experience dictates that when patients with concave stenosis undergo concave approach decompression, further lateral decompression of the facet joint is needed to expose the lateral edge of the nerve root due to anatomical malformations. Although the ODI results indicated that endoscopic decompression alone had similar efficacy for different types of patients, the VAS and JOA scores still indicated that a considerable proportion of patients with the Scce type had poor results following surgery and indicated that our surgical strategy was not suitable for treating patients with symptoms on the concave side, especially those with both symptoms and stenosis on the concave side. Notably, one patient with symptoms on the concave side had imaging results that showed convex stenosis in the spinal canal; this patient experienced little symptom relief after surgical decompression with the convex approach. Although the interpretability of individual results is limited overall, these findings may indicate that the presence of concave symptoms has a greater impact on prognosis than the choice of approach.

Current descriptions of degenerative scoliosis focus on its overall introduction, while studies of spinal stenosis and degenerative scoliosis are generally more focused on the treatment of spinal stenosis^17^. To determine the relationship between decompression level pairs and surgical outcomes, we need to understand the specific location of spinal stenosis in patients with scoliosis. At present, very few studies have examined the specific location of stenosis in patients. The statistical results of the current study suggest that the stenosis of degenerative scoliosis patients is not located inside the scoliosis curve as originally expected, especially near the top vertebra, but instead is mostly located at the edge of the end vertebra. The results showed that the difference in segmental positioning did not significantly affect the prognosis of the surgery, especially in terms of the maintenance and progression of the scoliosis angle and the pelvic balance of the patients. This finding also confirmed that endoscopic decompression alone is a reliable surgical technique for the treatment of patients with scoliosis and generally does not seriously exacerbate the degree of scoliosis within a certain follow-up period after surgery.

In this study, the complication rate of surgery was relatively low but notably differed from that of routine spinal canal decompression. Based on the analysis of a single patient with complications, we believe that surgical errors, especially judgment errors, are the main cause of complications. Due to the presence of spinal malformations, anatomical structures are not easily identified during decompression via endoscopy. Particular attention should be given to the following situations: 1. Care should be taken when the interlaminar space is very narrow on the laterally curved convex side due to overall degeneration and narrowing of the interlaminar space or the original imbricated structure is lost due to angulation of the interlaminar flexion. 2. Hyperplasia and hyperplasia of facet joints and surrounding hyperplastic tissues may bury the tail laminae deep inside the superior facet, which is easily hidden by the lower facet when it is exposed, increasing its likelihood of being mistakenly ground to the contralateral laminae. 3. When the head end of the laminae is ground for decompression, it is prone to deviate laterally, resulting in fracture of the lower articular process and severe residual low back pain, which may even force the patient to undergo additional fusion surgery. We believe that intraoperative X-ray localization of joint nodes is necessary, especially through orthographic localization, to ensure the timely detection and correction of surgical errors. 4. During convex decompression, the dura is more inclined to the dorsal side of the decompression side, and the surgical space between the dura and the ligamentum flavum is narrower. The dura is more likely to be damaged when the ligamentum flavum is peeled away to the central and dorsal sides of the spinal canal. The dura can only be discretely removed if the thickened edge of the ligamentum flavum presses against the dura ventrally.

In this study, the "SSS classification" system was established to classify patients, and surgical approach plans were formulated according to this classification. Most of the surgical plans formulated by this classification method were considered appropriate, and only when the symptoms of patients were located on the concave side did the endoscopic decompression plan used in the present study have a limited ability to alleviate symptoms. The concave side fusion approach adopted by Yang Hou may be an alternative surgical strategy ^18^, but further testing and study are needed. Due to the complexity of the study, our follow-up time for patients was at least 3 months; however, more long-term follow-up after surgery is needed in the future. In addition, with the further increase in the number of surgical patients, our surgical procedures have become more systematic and procedural and the technology has further matured, which may also have an impact on patient prognosis.

## Conclusion

Most surgical plans formulated using the "SSS classification" method were deemed appropriate, except when patients exhibited symptoms on the concave side. For these patients, the endoscopic decompression plan in this study demonstrated limited efficacy in symptom alleviation. A modified surgical strategy that enables concave decompression through a convex approach may prove beneficial; however, further investigation is warranted.

### Supplementary Information

Supplementary Information.

## Data Availability

All data generated or analyzed during this study are included in this published article and its supplementary information files.
